# Experimental Studies on Wave Interactions of Partially Perforated Wall under Obliquely Incident Waves

**DOI:** 10.1155/2014/954174

**Published:** 2014-09-01

**Authors:** Jong-In Lee, Young-Taek Kim, Sungwon Shin

**Affiliations:** ^1^Department of Marine and Civil Engineering, College of Engineering Sciences, Chonnam National University, 50 Daehak-ro, Yeosu, Jeonnam 500-749, Republic of Korea; ^2^River and Coastal Research Division, Korea Institute of Construction Technology, Goyang-si, Gyeonggi-do 411-712, Republic of Korea; ^3^Experimental Center for Coastal & Harbor Engineering, Chonnam National University, 50 Daehak-ro, Yeosu, Jeonnam 500-749, Republic of Korea

## Abstract

This study presents wave height distribution in terms of stem wave evolution phenomena on partially perforated wall structures through three-dimensional laboratory experiments. The plain and partially perforated walls were tested to understand their effects on the stem wave evolution under the monochromatic and random wave cases with the various wave conditions, incident angle (from 10 to 40 degrees), and configurations of front and side walls. The partially perforated wall reduced the relative wave heights more effectively compared to the plain wall structure. Partially perforated walls with side walls showed a better performance in terms of wave height reduction compared to the structure without the side wall. Moreover, the relative wave heights along the wall were relatively small when the relative chamber width is large, within the range of the chamber width in this study. The wave spectra showed a frequency dependency of the wave energy dissipation. In most cases, the existence of side wall is a more important factor than the porosity of the front wall in terms of the wave height reduction even if the partially perforated wall was still effective compared to the plain wall.

## 1. Introduction

Breakwaters have been built typically in the coast and harbor area to protect the coasts and properties from storms, to secure ship navigation, and to keep harbor tranquility. Different types of breakwaters, such as rubble-mound, caisson, perforated-wall caisson, and floating breakwaters, have been constructed by considering the site specific characteristics. A perforated-wall caisson breakwater was firstly proposed by Jarlan [1]. The perforated wall breakwater consists of an empty or partially-filled chamber between a perforated front wall and an impermeable rear wall. Since the efficiency of this type of breakwater with respect to wave reflection and energy dissipation in front of the structure was proven by laboratory experimental results, many researchers have proposed many different designs of perforated-wall structures. Boivin [2] tried to measure wave heights near the front of the perforated-wall caisson breakwater and proved the reduction of wave reflection by the structure. Huang et al. [3] introduced many previous studies on the hydrodynamics of different types of perforated or slotted structures and reviewed the analytical, numerical, and physical model studies in terms of the wave reflection, transmission, and force. They also discussed the numerical model results of the previous research work on a fully perforated breakwater, a partially perforated breakwater, a breakwater with multiple perforated walls, a perforated breakwater with a top cover, a perforated breakwater with a rock core, and a perforated breakwater with a horizontal porous plate in terms of the wave reflection, the wave transmission, and the wave forces on the structures. These kinds of perforated structures are typically used in Eastern Asia such as Korea, China, and Japan. Tanimoto and Yoshimoto [4] conducted a theoretical approach and laboratory experiment for the wave reflection characteristics of the partially perforated-wall caisson breakwater with a single chamber. Suh and Park [5] developed a numerical model to estimate the wave reflection coefficient from a perforated-wall caisson breakwater in regular wave cases based on the Galerkin-eigenfunction method. Suh et al. [6] also extended their model to simulate the wave reflection phenomena in irregular wave cases and verified their model by the data collected from the two-dimensional laboratory experiments. The study by Liu et al. [7] showed that the reflection coefficient dropped to a minimum when the ratio of the slit depth to the water depth is 0.5. Li et al. [8] studied the wave reflection characteristics of double-chambered perforated breakwaters considering obliquely incident regular waves. They proposed a theoretical model based on the eigenfunction expansion method. The model is capable of calculating the partially perforated-wall caissons with multiple chambers. The results showed that the reflection coefficient by obliquely incident waves varies with the widths and porosities of two chambers. As mentioned above, most of the previous studies on the perforated wall have been performed on the wave reflection characteristics in terms of numerical and analytical approaches under the normally incident wave conditions. However, the experimental studies for obliquely incident waves including stem wave effects are relatively scarce.

Perroud [9] first studied the stem wave, so called the Mach reflection, by generating a solitary wave to a vertical wall in the laboratory. Melville [10] found that the width and the height of a stem wave increases as a solitary wave propagates obliquely along a vertical wall from three-dimensional laboratory experiments. He conducted more detailed experiments on the Mach stem reflection to examine the stem wave evolution by generating an obliquely incident solitary wave. Yue and Mei [11] tried to describe the stem wave characteristics by using the nonlinear parabolic approximation. Liu and Yoon [12] extended the parabolic wave model in order to consider the varying water depth. Yoon and Liu [13] investigated the stem wave evolution due to obliquely incident cnoidal waves, by developing a parabolic approximation wave model from the Boussinesq equations. Their results showed that the normalized stem wave heights decrease as the nonlinearity of an incident wave increases and the incident angle decreases. They also showed that the stem wave width increases as the incident wave proceeds along the wall. Mase et al. [14] investigated stem wave evolution near a vertical wall due to obliquely incident random waves. The laboratory experiments and numerical simulations found that the normalized stem wave heights became larger as the incident wave angle increases or the nonlinearity of the incident waves decreases.

Lee et al. [15] studied experimentally and numerically the stem wave characteristics of monochromatic and random waves along the plain vertical wall. They compared the characteristics of wave transformation of monochromatic and random waves and the results showed that the wave heights increased near the beginning of the vertical wall and became constant after a certain distance for both monochromatic and random wave cases. They also used a parabolic approximation model and the REF/DIF to compare those results with the experimental data. In their studies, both models successfully reproduced stem wave evolution along the wall and the wave height variation normal to the wall.

In summary, most of previous studies on the perforated wall were focused on the theoretical and numerical approaches but the experimental studies were rare. Those studies were only performed about the wave reflection, the wave transmission, and the wave force. Other researchers investigated the stem wave characteristics from the analytical, numerical, and experimental approaches. However, all of their studies were done for the plain wall structure. Due to the complexity of the structural shape, it is extremely difficult to use analytical and numerical approaches on the three-dimensional hydrodynamic problems such as stem waves on the perforated wall. Moreover, there is no intensive study on the side wall inside the chamber of the perforated structures. Therefore, far more studies on the hydrodynamics of the partially perforated wall breakwater by obliquely incident waves are necessarily based on laboratory experiments under various wave conditions and the configurations of a front wall and a side wall. In this study, three-dimensional laboratory experiments were conducted to investigate the stem wave characteristics by obliquely incident waves under the different types of front and side walls. The next section describes the experimental setup, instrumentation, and configurations of the partially perforated wall structure. The third section shows the combination of test conditions including wave conditions (the wave height, the wave periods, and the incident wave angles). The study in this section includes three-dimensional wave characteristics on the vertical plain and partially perforated walls under the nonovertopping wave conditions. The fourth section shows the experimental results under various conditions according to the relative chamber widths, the types of front and side walls, and the wave conditions. The last section concludes and discusses the result of this study.

## 2. Experimental Setup

**Figure 1:**
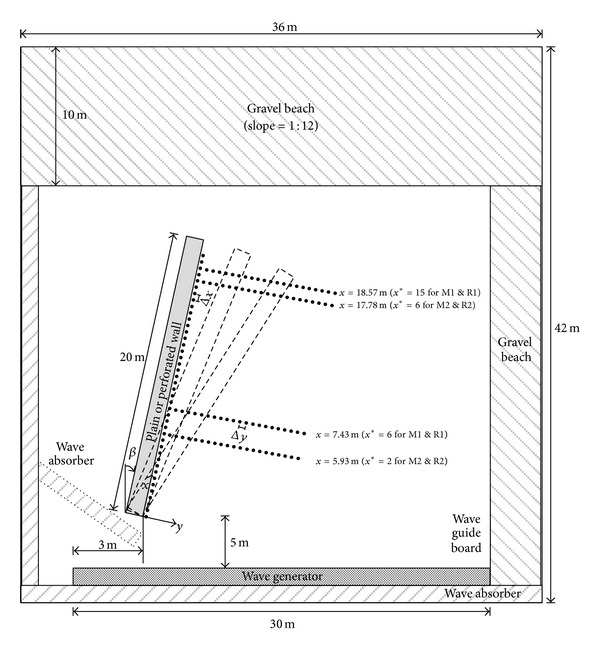
Experimental setup and measurement locations. Black dots are the capacitance type wave gages.

Three-dimensional laboratory experiments had been conducted in the wave basin at Korea Institute of Construction Technology, Republic of Korea. The dimension of the wave basin is 42 m long, 36 m wide, and 1.05 m deep. A multidirectional wave generator was installed in the basin and consists of 60 individual piston type wave paddles with the width of 0.5 m so as to generate multidirectional waves. Models of partially perforated and plain wall structures were installed 5 m from the wave generator and the angle of position (*β*) was changed from 10 to 40 degrees in order to give the effect of the incident wave angle variation. The total length of model structure was 20 m to allow the stem wave evolution over several times of wavelength as shown in Figure 1. Based on Table 3, the length of the model structure is longer than 8 times of the wavelength in case of R1 and M1 and longer than 3 times of the wavelength in case of R2 and M2. The configurations of model structures were carefully considered for realistic coastal structure, which is commonly used as a breakwater or a wharf structure in harbor area. The plain wall structure and the partially perforated wall structure were installed at the same location with the same dimension.  Table 1 introduces the types of model structures such as PW (plain wall), PV_NS (partially perforated wall with vertical-type slits at the front wall and without a side wall), PV_PS (partially perforated wall with vertical-type slits at the front wall and with plain side wall), and PV_VS (partially perforated wall with vertical-type slits at the front and side walls). Capacitance type wave gages were deployed along the wall (*x*-direction) and perpendicular to the wall (*y*-direction). The wave gages were installed 5 cm from the front wall and were deployed every 20 or 40 cm (Δ*x*) along the wall (*x*-direction). The gages were also deployed every 10 or 20 cm (Δ*y*) normal to the wall at* x* = 5.93, 7.43, 17.78, and 18.57 m from the left corner of the model in order to measure the spatial variations of wave heights in* y*-direction. The sampling rate of the wave gages was set to 20 Hz. Sloping gravel beaches were placed on all side walls of the basin and artificial wave absorbers were installed behind the wave paddles in order to minimize wave reflection from the basin wall.

**Figure 2:**
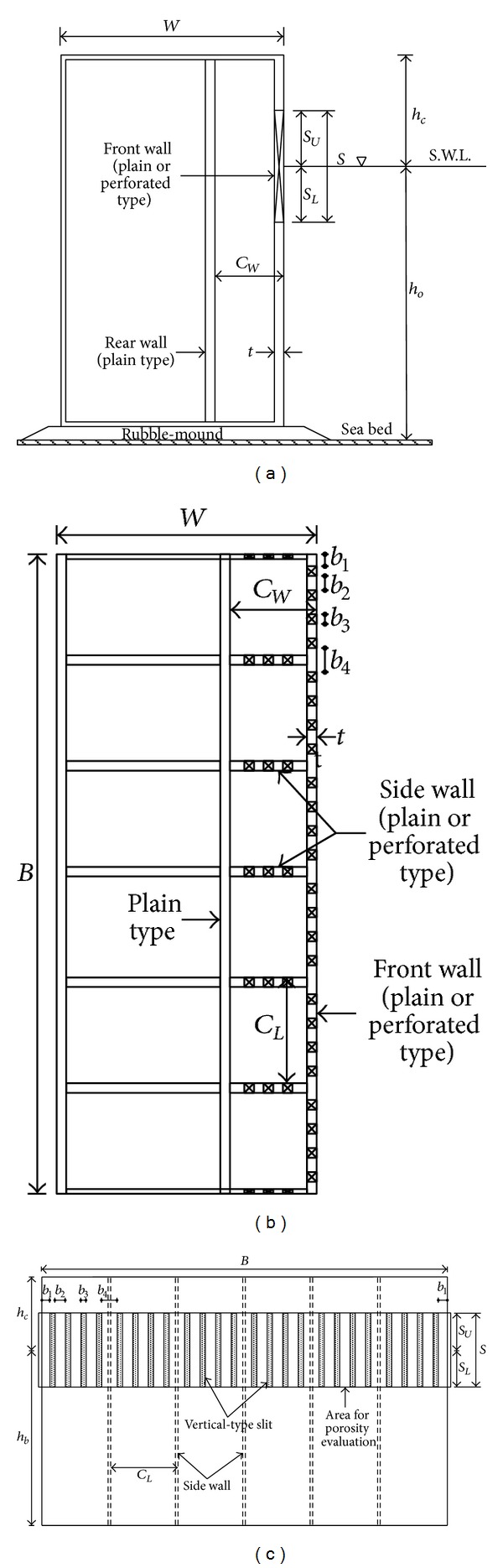
Schematic diagram of model structure. (a) Side view, (b) top view, and (c) front wall with vertical-type slit.

**Figure 3:**
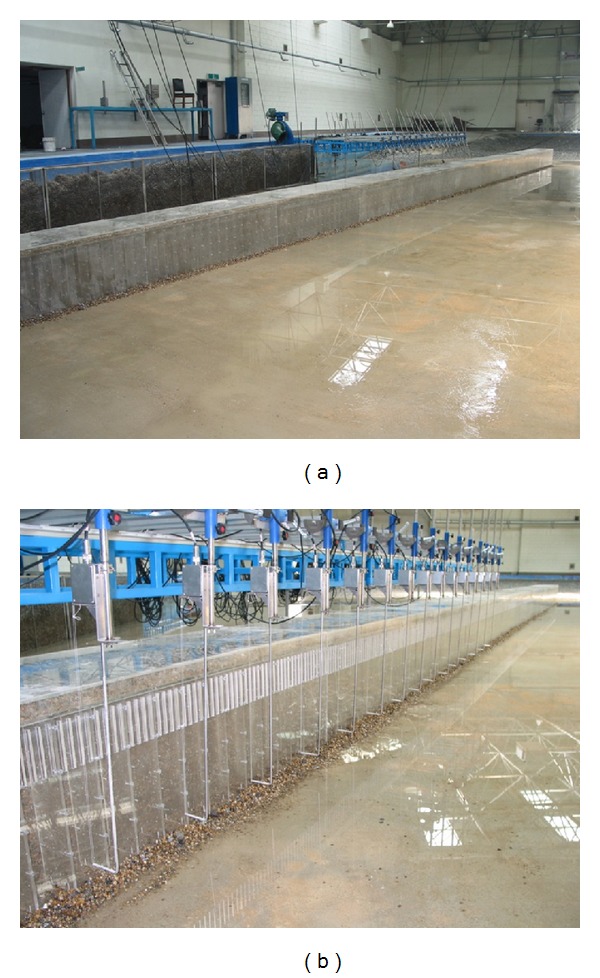
Photographs of model installation. (a) Plain wall (PW) and (b) partially perforated wall with vertical-type slits (PV_PS).

The detailed configuration of the partially perforated wall structure is shown in Figure 2 and Table 2. A box type vertical wall with a height of 55 cm and a width of 60 cm was installed on the rubble-mound with a height of about 5 cm. For the partially perforated wall, the front wall included the slit section with a vertical length of 13.7 cm and the still water level was located in slit section. Because the water depth was 45 cm in three-dimensional experiments, slit sections of the front wall vertically started at 38.8 cm and ended at 52.5 cm from the bottom of the basin considering *S*_*U*_ and *S*_*L*_ in Table 2. Therefore, the slit section allows most of the waves to pass through the partially perforated wall.  Figure 2(b) shows the top view of the partially perforated model. Both impermeable plain and perforated side walls were included inside the chamber every 12 cm in order to compare the characteristics of controlling obliquely incident waves.  Figure 2(c) shows the front views of the partially perforated wall structure that was used in the present study. The porosity of the vertical slit section in Figure 2(c) was set to approximately 29.2% by using the widths of *b*_1_~*b*_4_ for the vertical-type slit. Table 2 shows the dimension of all parameters in Figure 2.  Figure 3 shows the pictures of the plain wall (Figure 3(a)) and the partially perforated wall (Figure 3(b)) designed for the experiment in the present study. The structure is a single-chambered structure with a partially perforated front wall.

## 3. Test Conditions

A total of 16 different wave conditions were tested for the stem wave experiments in the experiments by considering the wave heights, the wave periods, the wave irregularity, and the incident wave angles as shown in Table 3. The cases of R1 and R2 stand for random wave conditions and M1 and M2 indicate monochromatic wave conditions. In the case of M1 and R1, the incident wave heights (*H*_0_ or (*H*_*s*_)_0_) of 0.03 m and the incident wave periods (*T*_0_ or (*T*_*s*_)_0_) of 0.9 seconds were generated by changing the incident wave angle (*β*) of 0 to 40 degrees. In this table, the subscript “0” denotes incident wave away from the structure and the subscript “*s*” denote the significant value of the random waves. So *T*_0_, *H*_0_, and *L*_0_ stand for the mean wave period, wave height, and the wavelength of the incident waves in case of monochromatic wave condition. On the other hand, (*T*_*s*_)_0_, (*H*_*s*_)_0_, and (*L*_*s*_)_0_ stand for the significant wave period, wave height, and wavelength of the incident waves in case of random wave condition. The calculated wavelengths (*L*_0_ and *L*_*s*_) were 1.238 m so that the relative chamber width, that is, the ratio of the chamber with to the wavelength (*C*_W_*), was 0.101. Similarly, in the case of M2 and R2, the relative chamber width was 0.042 based on the calculated wavelength. Random waves were generated by using Bretschneider-Mitsuyasu spectra that are widely used for simulating realistic ocean waves. Approximately 330 waves were used for data analysis such as the computation of wave heights and periods. Four different incident wave angles (10, 20, 30, and 40 degrees) were tested to examine the stem wave evolution characteristics due to the obliquely incident waves. Detailed measurement locations were also provided in Table 4. In this figure, x* indicates the relative length scale in *x*-direction normalized by the wavelength so x* of 2 means two times of the wavelength.

## 4. Results and Discussion

### 4.1. Relative Wave Heights along the Wall

**Figure 4:**
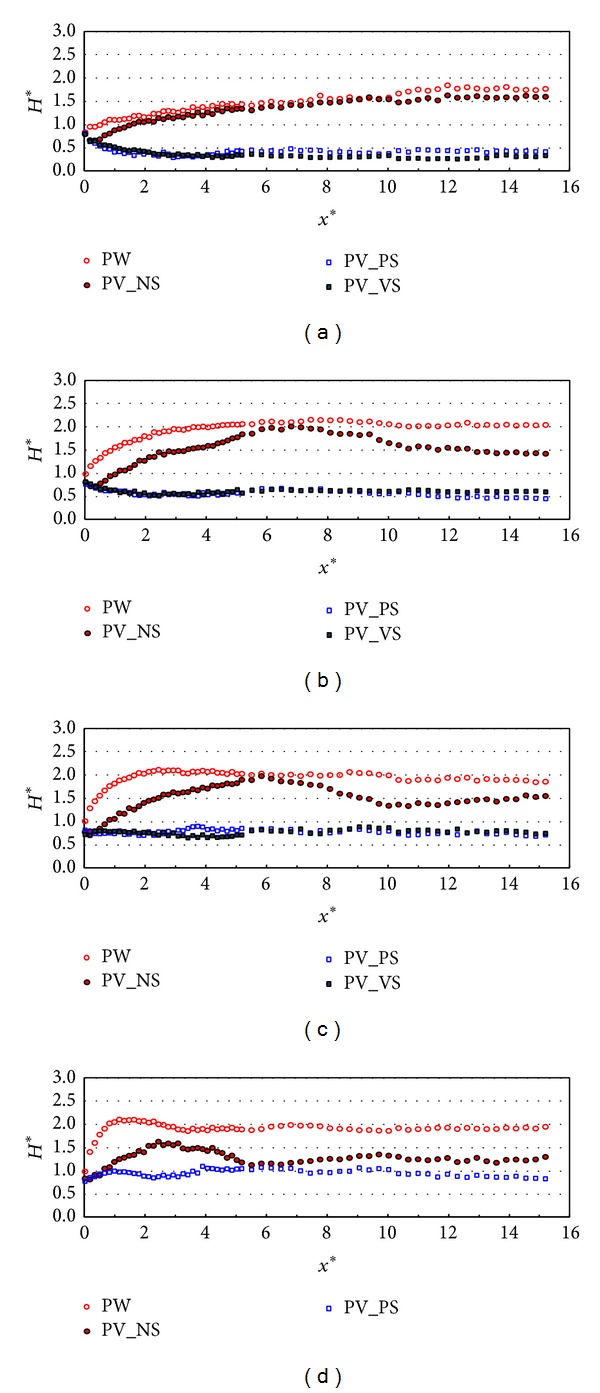
Relative wave heights along the wall (*x*-direction) by side wall types (TEST CASE: R1, *C*_W_* = 0.101). (a) *β* = 10°, (b) *β* = 20°, (c) *β* = 30°, and (d) *β* = 40°.

**Figure 5:**
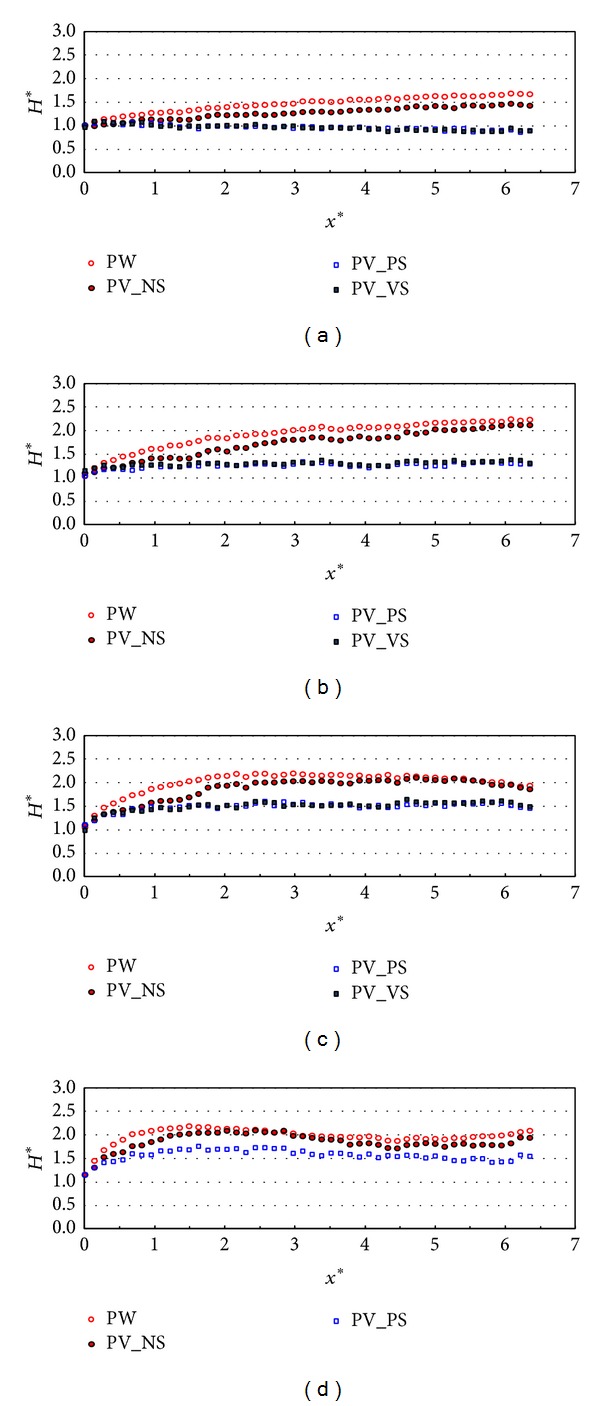
Relative wave heights along the wall (*x*-direction) by side wall types (TEST CASE: R2, *C*_W_* = 0.042). (a) *β* = 10°, (b) *β* = 20°, (c) *β* = 30°, and (d) *β* = 40°.

Figure 4 shows the relative wave heights along the wall (stem wave evolution) with different types of front and side walls in the test case of R1. Each figure shows the stem wave evolution under the different incident wave angles that range from 10 to 40 degrees when the relative chamber width (*C*_W_*) is 0.101. In the plain wall case (PW here in after), the relative wave heights (H* = *H*/*H*_0_ or H* = *H*_*s*_/(*H*_*s*_)_0_) along the wall show similar pattern to the previous studies [14, 15]. The relative wave heights along the wall gradually increase when the incident wave angles are small. As the incident wave angles increase, the relative wave heights along the wall rapidly reach a peak and converge. The results of the partially perforated wall without side wall (PV_NS here in after) show that the relative wave heights along the wall are similar to the results of PV_PS and PV_VS until x* is 0.5 but increase by showing a similar pattern of PW case. The figures also show that the difference between the PW and PV_NS cases in terms of the relative wave heights along the wall increases as the incident wave angles increase. The results of the partially perforated wall with the plain (PV_PS) and perforated side walls (PV_VS) show that the perforated wall with side walls controls stem wave evolution by 50~60% of those in both PW and PV_NS cases and shows better performance when the incident wave angle is small. However, there is no distinctive difference between the results of PV_PS and PV_VS in terms of stem wave evolution. Therefore, the partially perforated wall with the side walls is more effective than the partially perforated wall without the side walls with respect to the wave energy dissipation along the wall.  Figure 5 shows the relative wave heights along the wall with different types of front and side walls in the test cases of R2 as described in Table 4. The calculated relative chamber width (*C*_W_*) in this case is 0.042 which is smaller than the case in Figure 4. In this case, the relative wave heights along the wall in PW and PV_NS cases show a similar pattern but the results in PV_NS case are slightly smaller than those in PW case. The relative wave heights in case of PV_PS and PV_VS are similar to each other and reduced to less than 50% of those along the plain wall (PW). Over all, the relative wave heights along the wall in the case that the relative chamber width is smaller than that in Figure 4 are relatively larger under all the different configurations of partially perforated wall. Therefore, the relative chamber width can be another factor in the wave energy dissipation.

**Figure 6:**
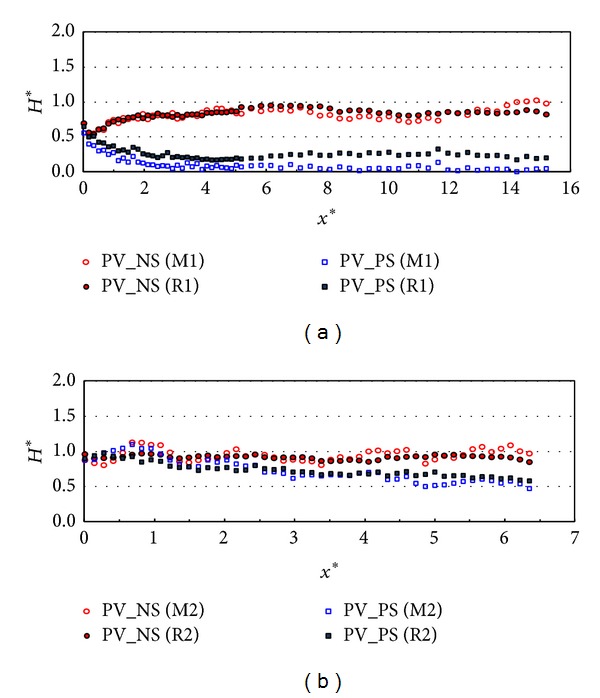
Relative wave heights along the wall for *β* = 0° (TEST CASE: PV_NS and PV_PS). (a) M1 and R1, *C*_W_* = 0.101 and (b) M2 and R2, *C*_W_* = 0.042.

In order to investigate the wave energy dissipation when the incident wave direction is parallel to the front wall, the relative wave heights along the wall (PV_NS and PV_PS) were plotted in Figure 6 when the incident wave angle is 0 degree.  Figure 6(a) shows the relative wave heights along the wall in both monochromatic (M1) and random (R1) wave cases when the relative chamber width is 0.101. The relative wave heights in PV_NS case start near 0.5 at x* being zero and increase up to one. In the case of PV_PS, the relative wave heights along the wall drop to 0.1~0.25 and converge to one near x* = 6. Therefore, the side wall affects the wave energy dissipation a lot more that the perforated front wall when the relative chamber width is 0.101 and the incident wave angle is zero. However, as shown in Figure 6(b), the wave energy dissipation is relatively small compared to the results in Figure 6(a) which is the case of larger relative chamber width. Also in the case of PV_PS, the wave heights are not reduced at x* = 0 compared to those in Figure 6(a) and gradually decrease up to 0.5. In both Figure 6(a) and Figure 6(b), the relative wave heights are reduced a little but more in the case of M1. The results in Figure 6 also show that the wave energy dissipation is more affected by the side wall than the front wall.

### 4.2. Relative Wave Heights Normal to the Wall

**Figure 7:**
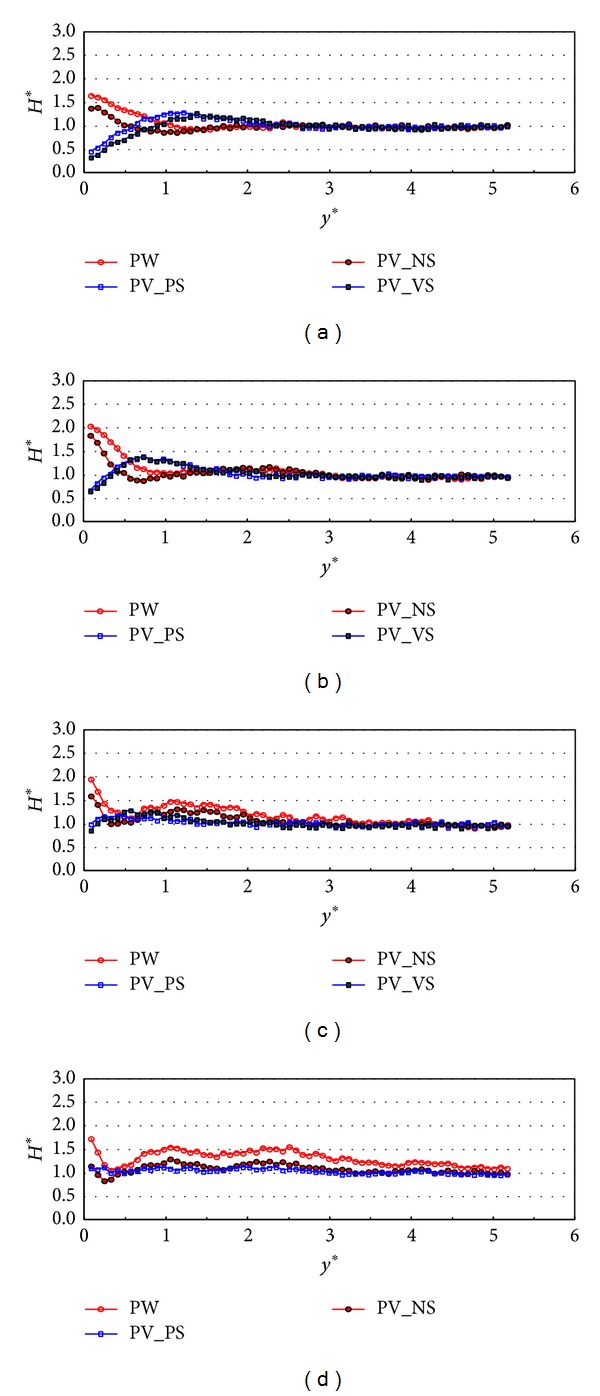
Relative wave heights normal to the wall (*y*-direction) by side wall types at x* = 6 (TEST CASE: R1, *C*_W_* = 0.101). (a) *β* = 10°, (b) *β* = 20°, (c) *β* = 30°, and (d) *β* = 40°.* y*∗ denotes the distance in* y* direction normalized by the significant wavelength (=* y*/*L*_*s*_).

**Figure 8:**
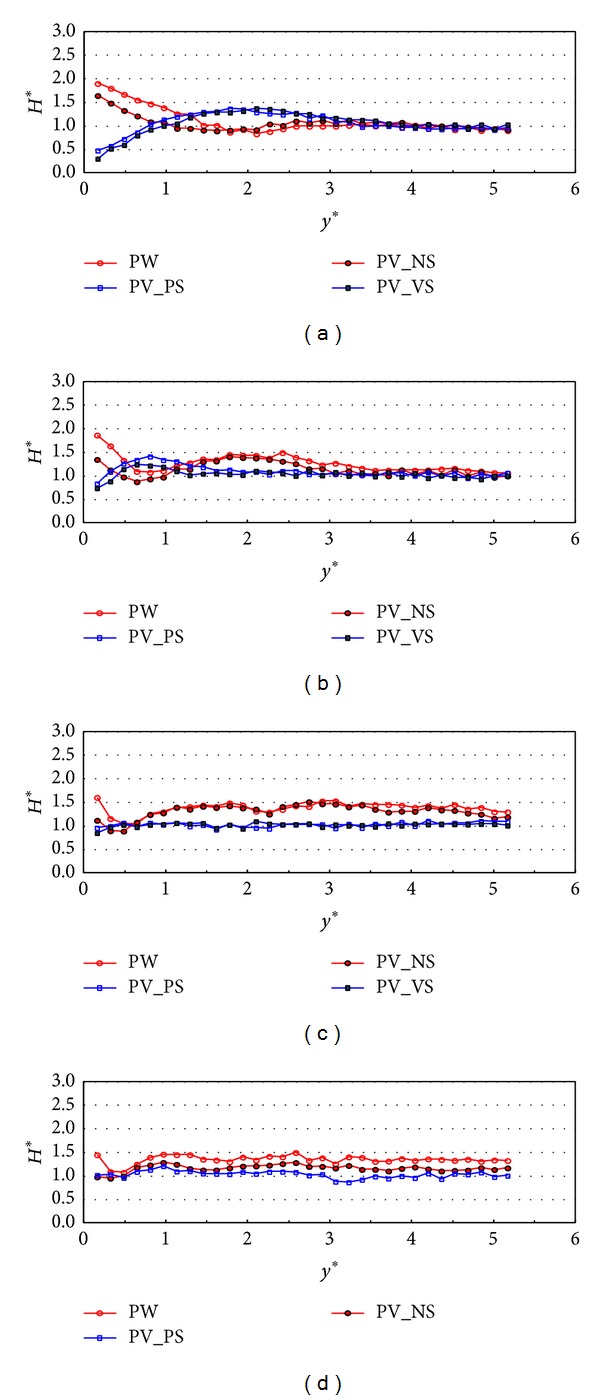
Relative wave heights normal to the wall (*y*-direction) by side wall types at x* = 15 (TEST CASE: R1, *C*_W_* = 0.101). (a) *β* = 10°, (b) *β* = 20°, (c) *β* = 30°, and (d) *β* = 40°.

Figures 7 and 8 show the experimental results under the wave condition of R1 which means that the relative chamber width (*C*_W_*) is 0.101. The comparison of the normalized wave height variations in the *y*-direction between PW, PV_NS, PV_Ps, and PV_VS cases at x* = 6 is shown in Figure 7. The cross-sectional variations (*y*-direction) of the relative wave heights on the different configurations of front and side walls were compared to each other with respect to the incident wave angles. Over all incident wave angles, the results in PW and PV_NS cases show similar patterns and the relative wave heights on PV_NS are 10~20% smaller than those on PW. The results show that the partially perforated front wall reduces wave energy by 10~20% mostly near the wall compared to the PW case. The wave energy dissipates more due to the perforated wall when the incident wave angle is large. These results show consistency with the relative wave height distributions along the wall as shown in Figure 4 because the relative wave height difference on PV_NS is a lot smaller than that on PW as the incident wave angle increase. In these two cases, the relative wave heights are at the maximum near the wall. On the contrary, in perforated wall cases with side walls (PV_PS and PV_VS), the relative wave height reaches almost minimum value near the wall and drops by 0.5 when the incident wave angles are 10 and 20 degrees. This seems to be because the perforated wall not only reduces the energy of waves but also makes a phase shift of wave reflection by passing the waves through the partially perforated wall. However, the relative wave heights near the wall mostly stay near one when the incident angles are larger than or equal to 30 degrees. The results in Figure 7 also show that, in wave energy reduction point of view, the perforated front wall performs better in larger incident wave angle and the side wall show a better performance in smaller incident wave angle. Therefore, for all four incident wave angles, the wave heights near the wall are a lot smaller in PV_PS and PV_VS cases than those in PW and PV_NS cases.  Figure 8 shows the relative wave height variations measured at x* = 15. The results of the relative wave height distributions show similar patterns to those in Figure 7 but the wave heights near the wall increase when the incident wave angle is 10 degrees and decrease when the incident wave angles are 30 and 40 degrees. These results are related to the stem wave evolution along the wall as shown in Figure 4. Figures 7 and 8 show that the stem wave width increases as x* increases.

**Figure 9:**
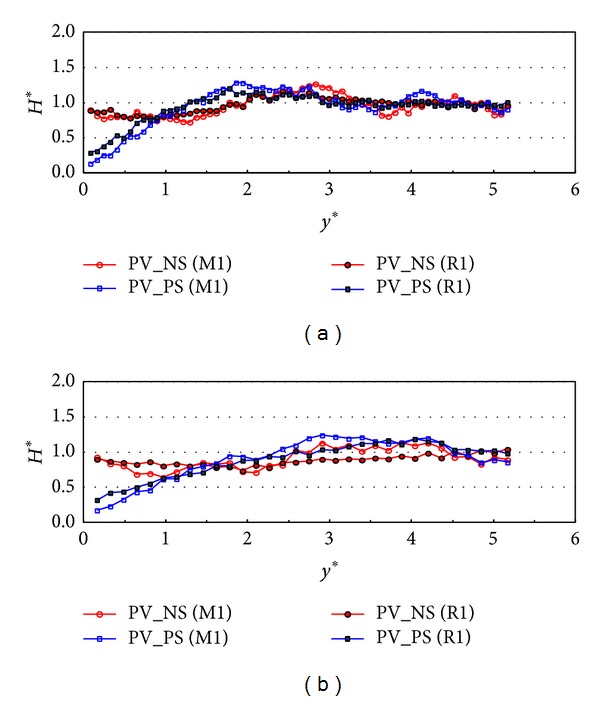
Relative wave heights normal to the wall (*y*-direction) for *β* = 0° (TEST CASE: M1 and R1, PV_NS and PV_PS, *C*_W_* = 0.101). (a) x* = 6 and (b) x* = 15.

Figure 9 shows the relative wave height distribution in *y*-direction under the wave conditions of M1 and R1 when the incident waves come parallel to the face of the wall. The data in Figures 9(a) and 9(b) describe the relative wave heights measured at x* = 6 and 15. In Figure 9(a), the relative wave heights in the case of PV_NS are a little bit less than one near the wall but they stay around one when y* is larger than two under both monochromatic and random wave conditions. However, in the case of PV_PS, the relative wave height drops to a minimum at the nearest location of the wall and increases up to one. The relative wave height variations in Figure 9(b) show similar trend to the results in Figure 9(a) but the range of the wave height reduction is longer when x* is 15. Therefore, the wave energy dissipates by 70~90% near the wall and the dissipation range in* y*-direction increases as x* increases and the side walls are much more effective than the perforated front wall in terms of wave energy dissipation.

**Figure 10:**
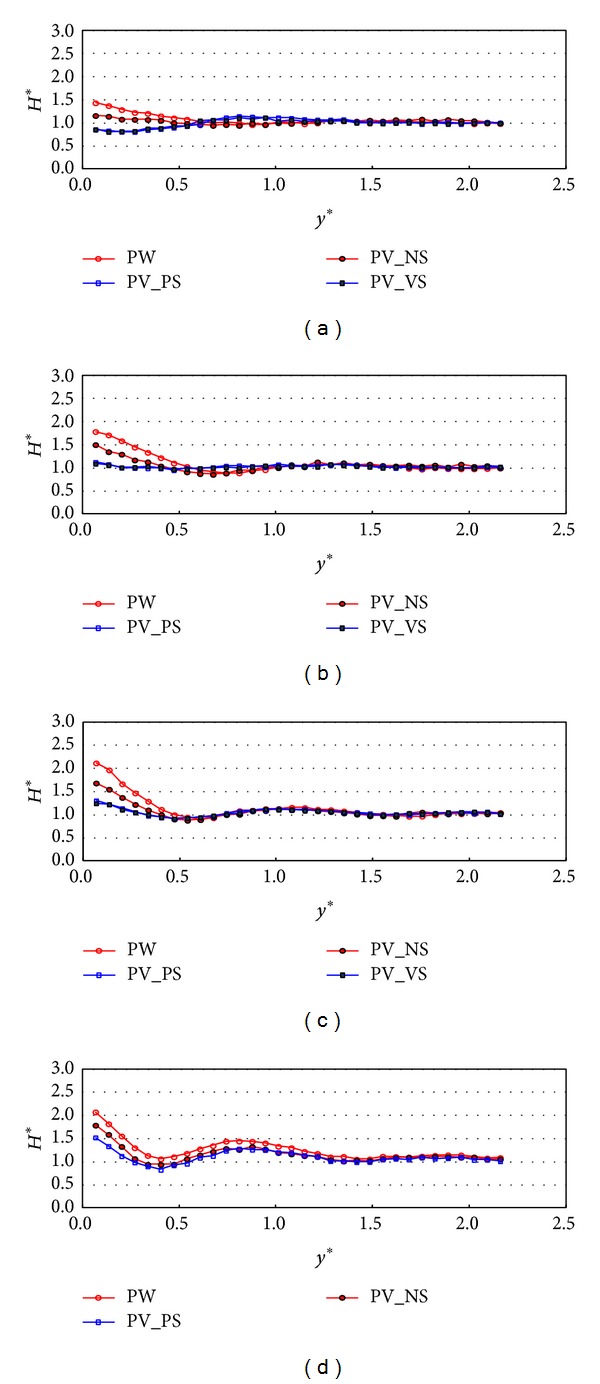
Relative wave heights normal to the wall (*y*-direction) by side wall types at x* = 2 (TEST CASE: R2, *C*_W_* = 0.042). (a) *β* = 10°, (b) *β* = 20°, (c) *β* = 30°, and (d) *β* = 40°.

**Figure 11:**
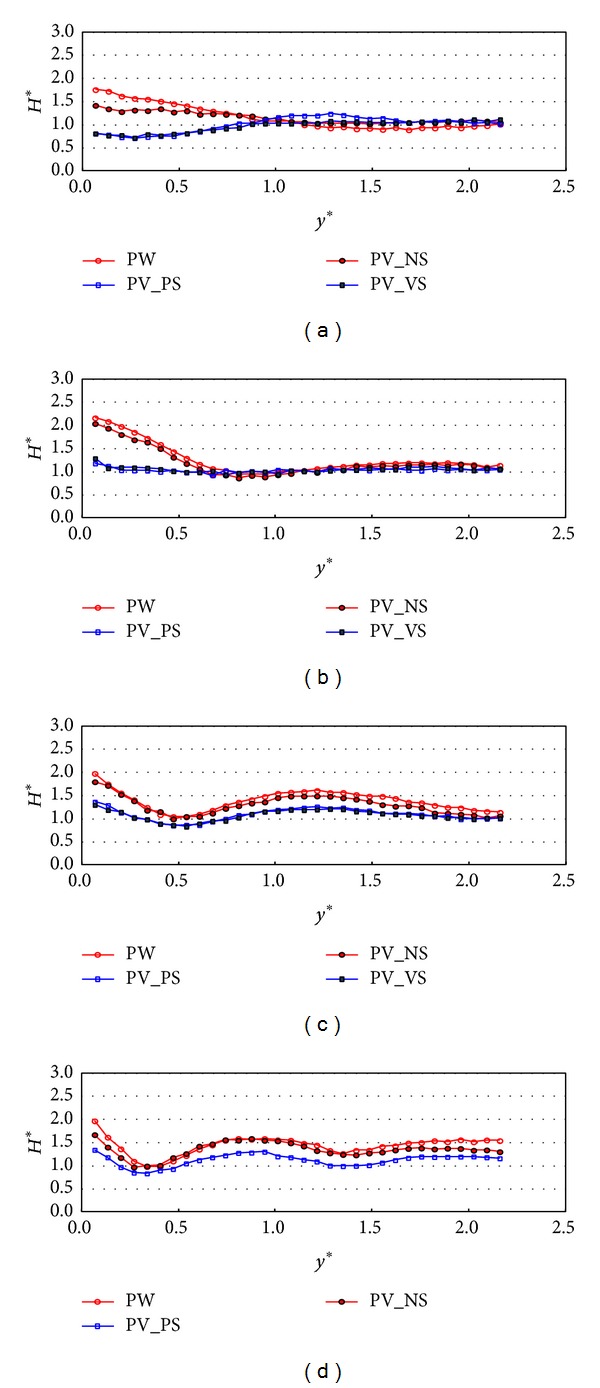
Relative wave heights normal to the wall (*y*-direction) by side wall types at x* = 6 (TEST CASE: R2, *C*_W_* = 0.042). (a) *β* = 10°, (b) *β* = 20°, (c) *β* = 30°, and (d) *β* = 40°.

Figure 10 shows the cross-sectional variations (*y*-direction) of relative wave heights for random waves with respect to different incident wave angles at x* = 2 when the relative chamber width is 0.042, which is nearly a half time smaller than the case of Figure 7. There are also some kinds of phase shifts as well as energy dissipation in between the plain wall and partially perforated wall with side walls cases. The phase shifts are more clearly shown when the incident wave angle is small and show a slightly different pattern of fluctuation width to the results in Figure 7 so that the relative wave heights near the wall are larger than one as the incident wave angle becomes larger than 10 degrees. The results of PV_NS in Figure 10 reduce the wave energy by 10~20% near the wall and follow the pattern of PW case. On the other hand, PV_PS and PV_VS reduce the relative wave heights by 30 to 40% of those in the PW and PV_NS cases and the energy dissipates a lot more in the smaller incident angle cases. Similar to the results in Figure 7, the effect of side wall is larger than that of the perforated front wall with respect to the wave height reduction in* y*-direction. This seems to be because the side wall induces the multireflection effects that may dissipate more wave energy.  Figure 11 shows the relative wave height distribution with the same conditions as Figure 10 but the data were collected at x* = 6. In smaller incident wave angle (10 and 20 degrees), the relative wave heights near the PW and PV_NS are larger than those in Figure 10 due to the stem wave evolution. PV_PS and PV_VS reduce the relative wave heights by 30 to 50% of those in the PW and PV_NS cases. Again, there is no distinctive difference between the PV_PS and PV_VS cases in terms of the relative wave height variations in* y*-direction. Figures 7 and 11 show the relative wave height distributions at the relative position in *x*-direction (x* = 6). In both figures, the amount of the wave energy dissipation near the wall is larger in Figure 7. Therefore, the relative chamber width of 0.101 also contributes the wave energy dissipation more than that of 0.042.

**Figure 12:**
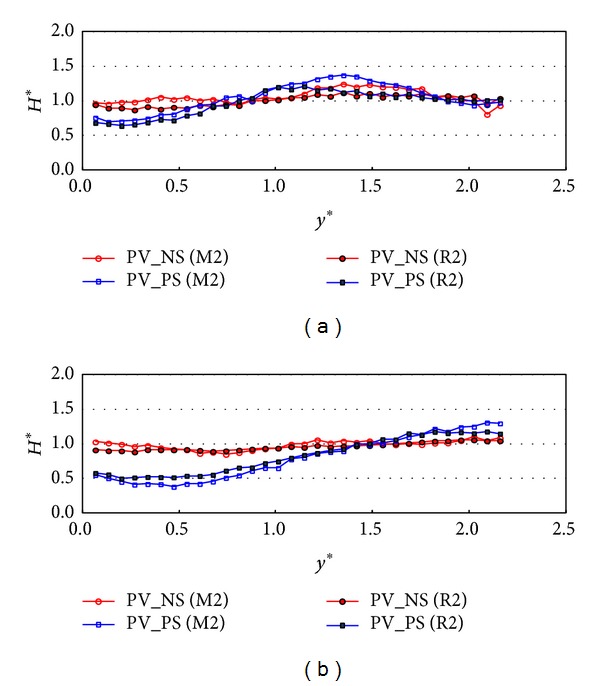
Relative wave heights normal to the wall (*y*-direction) for *β* = 0° (TEST CASE: M2 and R2, PV_NS and PV_PS, *C*_W_* = 0.042). (a) x* = 2 and (b) x* = 6.

Figure 12 shows the relative wave height distribution in* y*-direction under the wave conditions of M2 and R2 when the incident wave angle is zero and the relative chamber width is 0.042 which is similar to Figure 9 but the relative chamber width is small. The data in Figures 12(a) and 12(b) were measured at x* = 2 and 6. Over all, the results in Figure 12 follow a similar trend to the results in Figure 9 but the amount of the wave energy dissipation is less than that in Figure 9. The widths of the wave energy dissipation in* y*-direction (y* ≈ 0.8 in Figure 12(a) and y* ≈ 1.5 in Figure 12(b)) are similar to those in the case of the relative chamber width of 0.101 as shown in Figure 9. In this incident wave direction, the side wall and the chamber width contribute to the wave energy dissipation near the wall.

### 4.3. Spectral Comparison of Waves Based on the Front Wall and Side Wall Types

**Figure 13:**
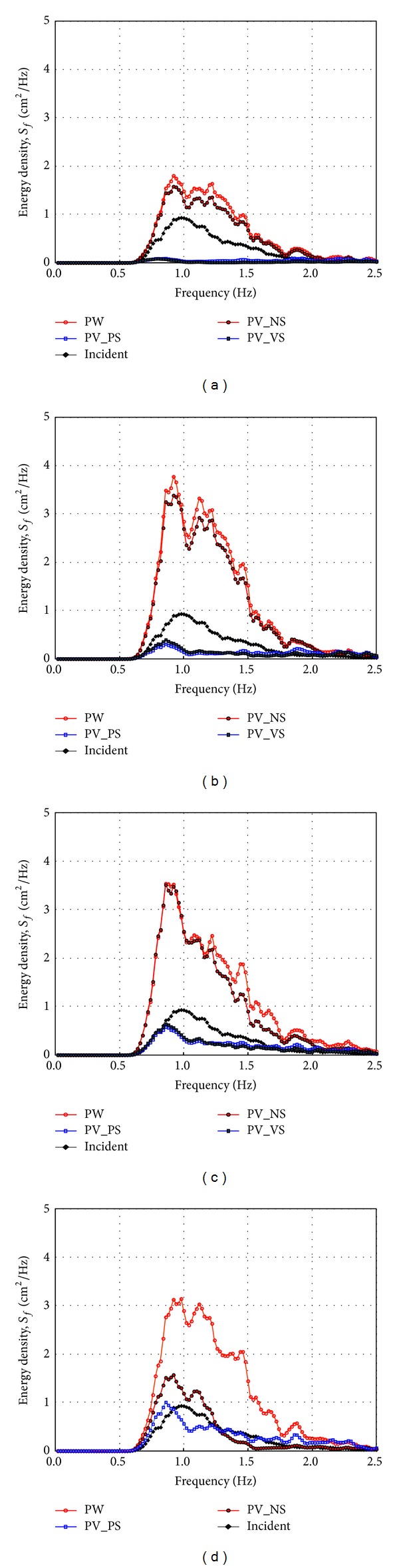
Measured frequency spectrum by side wall types at x* = 6.46 (TEST CASE: R1, PW and PV_NS and PV_PS and PV_VS, *C*_W_* = 0.101). (a) *β* = 10°, (b) *β* = 20°, (c) *β* = 30°, and (d) *β* = 40°.

Figure 13 shows the frequency spectra of free surface elevations in front of the four different configurations of the front and side walls when *C*_W_* is 0.101 and the incident wave angles are 10~40 degrees. As already shown in Figure 4, the energy spectra in the PW and PV_NS cases are larger than those of incident waves at x* = 6.46. In contrast, the energy spectra of the water surface elevations, measured in front of the PV_PS and PV_VS, are smaller compared to those of the incident wave and the energy dissipated more when the incident angle is small. These energy spectra also show the frequency dependence of energy dissipation whereas Figure 4 shows only the wave height ratio. Especially, it is clearly shown that the energy near the peak frequency dissipates a lot so that the peak frequencies of the PV_PS and PV_VS cases are shifted. In Figure 13(a), the wave energy dissipates in almost whole frequency range when the incident wave angle is 10 degrees but, as the incident wave angle increases, the amount of the energy dissipation decreases especially in lower frequency range. Other than Figures 13(a)–13(c), the wave energy also dissipates a lot in PV_NS case. This result is consistent with the result in Figure 4(d) that the relative wave heights rapidly drop to one when x* is near six.

**Figure 14:**
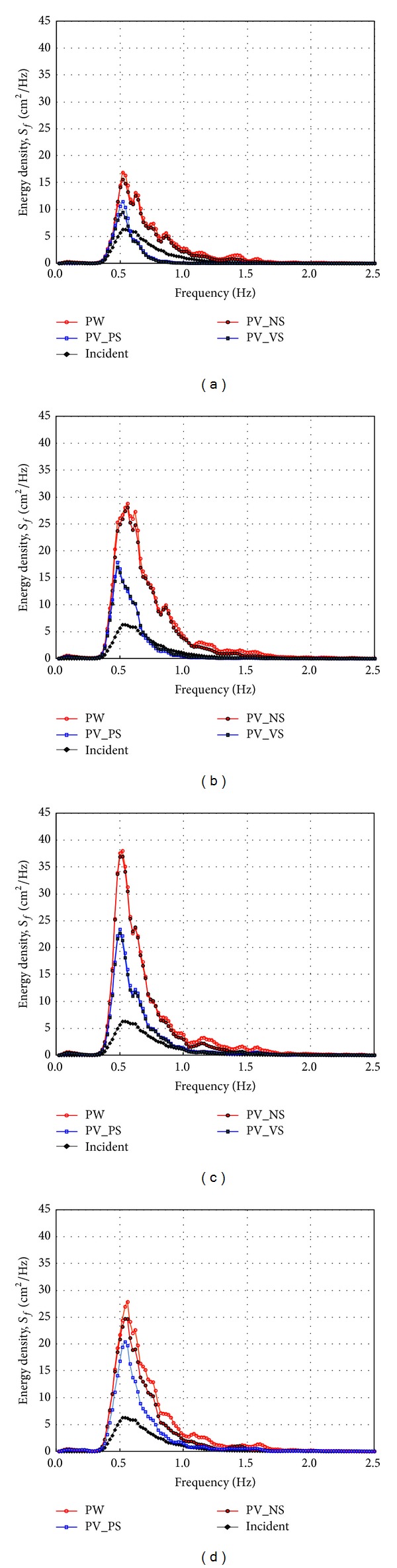
Measured frequency spectrum by side wall types at x* = 5.40 (TEST CASE: R2, PW and PV_NS and PV_PS and PV_VS, *C*_W_* = 0.042). (a) *β* = 10°, (b) *β* = 20°, (c) *β* = 30°, and (d) *β* = 40°.

Figure 14 shows the wave spectra when the relative chamber width (*C*_W_*) is 0.042. The wave energy dissipation is less than the results in Figure 13 but the PV_PS and PV_VS still reduce the wave energy compared to the results of the PW and PV_NS. Both Figure 13 and Figure 14 indicate that the PV_PS and PV_VS reduce the wave energy more than the PW and PV_NS do and there still is a little frequency dependency according to the incident wave angle.

### 4.4. Effects of the Relative Chamber Width (*C*_W_*)

**Figure 15:**
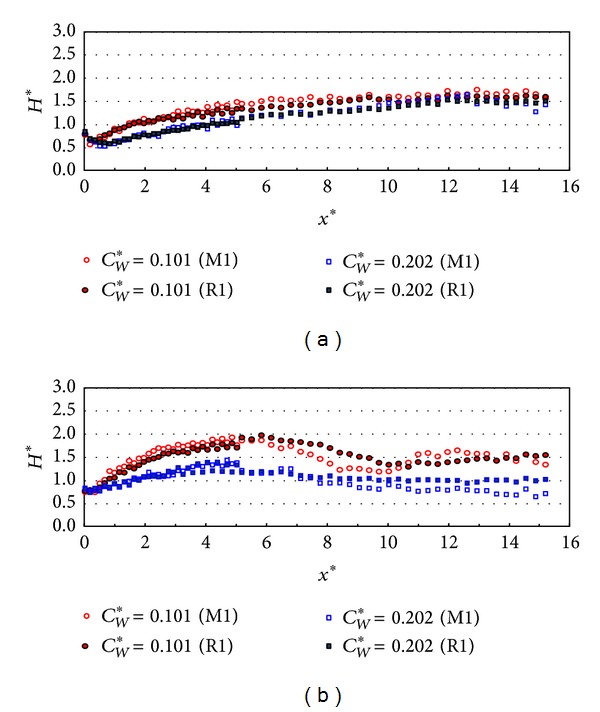
Relative wave heights along the wall (*x*-direction) by chamber width (TEST CASE: M1 and R1, PV_NS). (a) *β* = 10° and (b) *β* = 30°.

**Figure 16:**
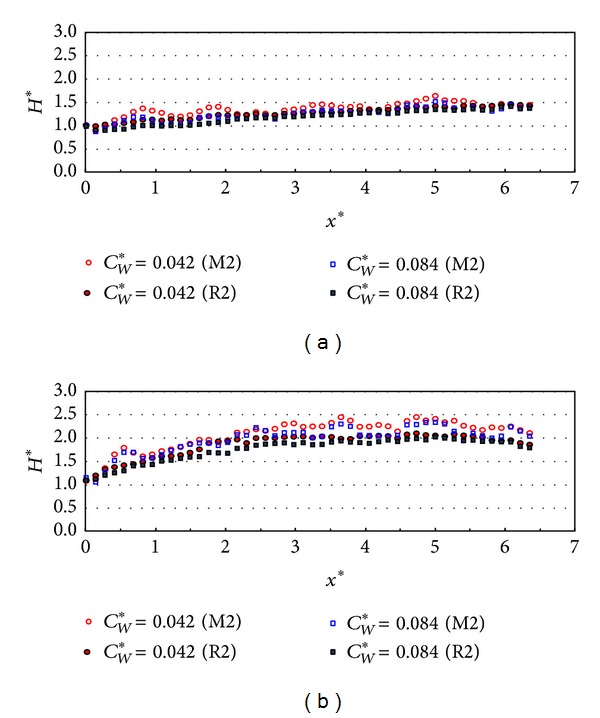
Relative wave heights along the wall (*x*-direction) by chamber width (TEST CASE: M2 and R2, PV_NS). (a) *β* = 10° and (b) *β* = 30°.

In order to investigate the effect of the relative chamber width on the wave energy dissipation, the experimental data are plotted according to the relative chamber widths of 0.042~0.202 in Figures 15 and 16.  Figure 15 shows the relative wave height distribution in *x*-direction when the relative chamber widths are 0.101 and 0.202 in the case of PV_NS. In the case of smaller incident wave angle (10 degrees), the relative wave heights along the wall when the relative chamber width (*C*_W_*) is 0.202 are smaller by 30% than the results with the relative chamber width of 0.101.  Figure 15(b) shows that the relative wave heights with the relative chamber width of 0.202 are smaller by 40% compared to the results with the relative width of 0.101. Therefore, the results in Figure 15 show that the chamber width influence more wave energy dissipation in larger incident wave angle when the relative chamber width is 0.202. Over all, the monochromatic wave cases show a little more wave energy dissipation than the random wave cases.

Figure 16 shows the relative wave heights along the wall with the relative chamber widths of 0.042 and 0.084. When the incident wave angle is 10 degrees as shown in Figure 16, the wave energy dissipation is relatively small compared to the results in Figure 15. There is no significant effect of the relative chamber width on the wave energy dissipation in this case.

## 5. Conclusions

Laboratory experiments were conducted in a three-dimensional wave basin to investigate the wave energy dissipation performance of different types of front and side walls. The four different configurations of the wall types (PW, PV_NS, PV_PS, and PV_VS) were tested to understand their effects on the stem wave evolution and the wave energy dissipation. Several different wave conditions of both monochromatic and random waves were used to figure out the effects of relative chamber widths. Incident wave angles ranging from 0 to 40 degrees were also considered to figure out the stem wave evolution along the wall with different types of front and side walls. Wave spectra in all cases were analyzed to investigate the frequency dependency in terms of the wave energy dissipation.

The following conclusions are drawn based on the experimental results in the present study.

1. The partially perforated wall structure (PV_NS, PV_PS, and PV_VS) reduces the stem wave evolution along the wall more effectively compared to the plain wall structure (PW), especially in the case of the smaller wave incident angle. Among the different types of partially perforated walls, the perforated walls with side walls (PV_PS and PV_VS) showed a better performance in the wave height reduction compared to the structure without the side wall. However, there is no significant difference of the wave height reduction whether the side wall is impermeable or partially permeable. Moreover, the relative wave heights along the wall are small when the relative chamber width (*C*_W_*) is 0.101 compared to the results from the relative chamber width of 0.042.
2. Based on the experiments of wave height distribution in the normal direction to the wall, the structural types of PV_PS and PV_VS also control wave heights near the front wall compared to the PW and PV_NS cases. It seems that the effects of the side wall, the perforation, and the relative chamber width are combined in reducing the wave heights near the front of the wall. Especially in the small incident wave angles, the side walls give strong impact on the wave height reduction near the wall. Therefore, this type of structure is also applicable to wharf structure.
3. The wave spectra show that the partially perforated walls with side walls (PV_PS and PV_VS) reduce the wave energy and the major reduction is near the peak frequency of incident wave spectra when the relative chamber width is 0.101. However, in the smaller value of the relative chamber width of 0.042, the partially perforated walls with side walls somehow reduce the wave energy compared to the PW and PV_NS cases but the amount of reduction is smaller than the case of larger relative chamber width.
4. The wave energy dissipation was also found when the incident wave angle is zero which the waves come parallel to the wall face. The results also prove that the side walls are more effective than the front wall type in terms of the wave energy dissipation.
5. Additional tests of the relative wave heights along the wall (PV_NS) were conducted by changing the relative chamber width from 0.042 to 0.202. The results show that the larger relative chamber width reduces the wave energy more than the smaller one does within the range of chamber width in the present study. However, in the wave randomness point of view, there is no big difference of wave height variations between the monochromatic and random waves regardless of the chamber width, the wave condition, and the incident wave angle.

So far, no experimental study as well as numerical simulations has been performed for the wave height distribution to the perforated wall structure with side walls in the condition of obliquely incident waves. However, this study newly found that the existence of side wall inside the chamber reduces the wave energy effectively. Therefore, in most cases, the existence of side wall is more important factor than the porosity of the front wall even if the partially perforated wall is still effective compared to the plain wall. The results in this study can be applied to design the breakwater or wharf structure to protect coastal and harbor area and to enhance the ship operation and navigation. This data set can also contribute to the numerical model verifications and improvements.

## Figures and Tables

**Table 1:** Types of model structure.

Case ID (model structure)	Front wall type	Side wall type
PW	Plain wall	—
PV_NS	Partially perforated wall with vertical-type slit	None
PV_PS	Partially perforated wall with vertical-type slit	Plain wall
PV_VS	Partially perforated wall with vertical-type slit	Partially perforated wall with vertical-type slit

**(a):** 

*W*	*B*	*h*_0_	*h*_*C*_	*S*	*C*_*W*_	*C*_*L*_	*t*	*S*_*U*_	*S*_*L*_
60.0	78.0	45.0	15.0	13.7	12.5	12.0	1.0	7.5	6.2

**(b):** 

Front wall with vertical-type slit (porosity *≒* 29.2%)			
*b*_1_	*b*_2_	*b*_3_	*b*_4_
1.25	2.00	0.88	2.50

**Table 3:** Wave and test conditions in the experiments.

Case ID (wave)	Wave period, *T*_*0*_ and (*T*_*S*_)_0_ (sec)	Wave height, *H*_*0*_ and (*H*_*S*_)_0_ (m)	Wavelength, *L*_*0*_ and (*L*_*S*_)_0_ (m)	Incident angle, *β* (°)	Water depth, *h*_*0*_ (m)
R1, M1	0.9	0.03	1.238	10, 20, 30, 40	0.45
R2, M2	1.6	0.06	2.963		

**Table 4:** The locations of wave gages.

Cases	*x*-direction (along the front wall)		*y*-direction (normal to the wall)	
			at x* = 2 & 6	at x* = 15
R1, M1	*x* = 0 m~6.4 m (Δ*x* = 0.2 m)	*x* = 6.4 m~18.8 m (Δ*x* = 0.4 m)	*y* = 0.1 m~6.4 m (Δ*y* = 0.1 m)	*y* = 0.2 m~6.4 m (Δ*y* = 0.2 m)
R2, M2	*x* = 0 m~18.8 m (Δ*x* = 0.4 m)		*y* = 0.2 m~6.4 m (Δ*y* = 0.2 m)	—

x* denotes distance in *x*-direction normalized by the significant wavelength (=*x*/Ls).
